# Supplementary material for: Relationship between urban morphology and land surface temperature—A case study of Nanjing City
Source: PLoS One. 2022 Feb 9;17(2):e0260205. doi: 10.1371/journal.pone.0260205 (PMC8827472; doi:10.1371/journal.pone.0260205)
Supplement: Attachment — Submitted filename: NJC2(authors)-with comments.docx [file pone.0260205.s001.docx]

Relationship between urban morphology and land surface temperature – a case study of Nanjing City

Shusheng Yin ^a,*^  , Jiatong Liu ^a^, Zenglin Han ^a,b^

^a^ college of Geography science, Liaoning Normal University,Dalian,116029,China

^b^ Center for studies of marine economy and sustainable development, Liaoning Normal University, Dalian,

116029, China

Abstract：

This study investigated the relationship between urban form and land surface temperature (LST) using the Multi-access Geographically Weighted Regression (MGWR) model. A case study on Nanjing City was conducted using building data, point-of-interest (POI) data, land use data, remote sensing data, and elevation data. The results show that the MGWR model can reveal the influence of different variables on LST, with a superior fitting effect over the geographically weighted regression model. LST in Nanjing exhibits a significant spatial differentiation, and the distribution of LST hotspots is spatially consistent with the level of urban construction. In terms of the two-dimensional landscape pattern, LST decreases with altitude and increases with POI. In terms of the three-dimensional structure, building height has a positive correlation with LST. POI, urban roads, and urban buildings positively affect LST, while urban green space and altitude negatively affect LST. The results of this study were verified against existing findings. The LST of areas with high-rise and super high-rise buildings is lower than that of areas with mid-rise building, which can be attributed to the large number of shadow areas formed by high-rise and super high-rise buildings. A similar phenomenon was also observed between areas with medium- and high-density buildings.

Keywords: LST; MGWR; Urban form; Influencing factors

1. **Introduction**

With the continuous expansion and increase of the scale and number of cities in China, the boundary between urban and rural areas is becoming increasingly blurry. The urbanization rate of China's permanent population reached 60.60% in 2019^[1]^.The continuous expansion of the scale of related industrial activities (Liu, 2017; Wang and Wang, 2011) is driving economic growth, improving the employment environment, and increasing the income of residents (Guo et al., 2020; Li et al., 2020; Tang et al., 2020). However, this expansion has also led to negative effects on the quality of human settlements, social and economic development (Chen et al., 2020; Gu et al., 2019; Liu and Leng, 2020; Mashhoodi et al., 2020; Zhou et al., 2017). In particular, the urban heat island effect, especially urban heat waves associated with global warming, can increase the vulnerability of populations to various health issues, such as heatstroke, and even death. Therefore, improving the urban thermal environment has become the focus of relevant scholars and institutions. To measure the quality of the urban thermal environment and mitigate the negative effects, it is necessary to analyze the spatiotemporal pattern of the thermal environment and its influencing factors. For this purpose, various indicators have been developed. Among them, Land Surface Temperature (LST), a basic parameter in the fields of meteorology and ecological changes (Hu et al., 2020; Zhang et al., 2020), has become an important indicator (Cheng et al., 2019; Stewart, 2011; Xue et al., 2017), facilitating detailed analyses.

The relationship between urban landscape patterns, such as land use type and blue-green space (Oke, 1981), and surface temperature has received extensive attention worldwide. At present, researchers in the field of LST studies are focusing on the following aspects: (1) The impact of the two-dimensional urban landscape pattern on LST, analyzing the impact of different land use types on LST from the perspective of land use type and land use type transformation (Liu et al., 2019; Zhao et al., 2016; Zhou and Tian, 2020); (2) Statistical analysis of the mathematical relationship between surface factors and LST using indicators, such as normalized difference vegetation index, normalized difference moisture index, normalized difference built-up index, and building density (BD) (); (3) Correlation between the three-dimensional structure and LST based on indicators, such as building height (BH), floor area ratio (FAR), and sky view factor (SVF). It is worth noting that the process of urbanization involves a contradiction between population concentration and limited supply of construction land, which further leads to the rapid expansion of cities in the two-dimensional direction and the continuous increase of BH (Qin et al., 2020). However, most studies investigated the relationship between surface factors and LST through linear regression analysis, ignoring the spatial autocorrelation and spatial information between the two. In addition, research on the relationship between urban form and LST mostly focus on a single factor of urban form (two-dimensional or three-dimensional), especially the two-dimensional form. The lack of multi-factor analysis combined with the two-dimensional landscape pattern and three-dimensional structural form makes it difficult for existing studies to further explore the impact of urban spatial differences on urban thermal environment. In addition, the urban spatial planning system has undergone major changes, and the three-dimensional urban structure is undergoing significant changes. Therefore, the existing approach of analyzing the urban thermal environment needs to be clarified and the two-dimensional landscape and three-dimensional space of urban landscapes need to be optimized.

This study aims to comprehensively explore the relationship between urban form (two-dimensional landscape pattern and three-dimensional structural form) and LST. For this purpose, the Multi-scale Geographically Weighted Regression (MGWR) model, a mono-window algorithm, was used to quantitatively analyze the spatial pattern of related surface factors in each local climate zone of Nanjing on LST. With this approach, this study addresses the lack of multi-factor analysis to a certain extent. The findings will provide support for regulating the urban thermal environment and improving the quality of urban human settlements.

2. **Study area and data sources**

### 2.1 Overview of the study area

Nanjing is located at 31°14'N-32°36'N, 118°22'E-119°14'E. It is a low altitude area with a humid subtropical climate (Cfa). According to meteorological records, the extreme daily maximum temperature in Nanjing is 43.0 ℃. It has 11 districts with a total area of 6587 km^2^ and a built-up area of 817 km^2^. As of 2019, permanent residents account for a population of 8.5 million and an urbanization rate of 83.2%. The increasing concentration of population and amount of industrial activities are exacerbating the urban heat island effect (Fig.1).

Fig.1 Location of the study area

### 2.2 Data source and processing

In this study, building data, POI data, land use data, remote sensing data and elevation data were selected. The data sources and descriptions are shown in Table 1. BH and BD reflect the concentration of buildings in the vertical and horizontal directions, respectively. To a certain extent, the two have the greatest impact on the three-dimensional structure of the city, and the degree of LST has the most direct effect (Oke et al., 1981; Zhou et al., 2020). According to the 2019 Unified Standards for Civil Building Design, BH is divided into 3 types: low-rise civil buildings (≤27 m), high-rise civil buildings (27–100 m), and super high-rise buildings (>100 m). Considering the findings of existing research and the status quo of the research area, with 20% and 40% as the boundary, BD was divided into three types: low density, medium density, and high density (Wan et al., 2010). POI data were obtained through Baidu Map API, and the data were cleaned and selected for nuclear density analysis. LST data were obtained through inversion using ENVI5.3 software with Landsat 8 products, which were geometrically corrected. Urban green space and aspect were obtained from digital elevation data using the ArcGIS 10.7 surface analysis function.

Table 1 Data source and description

| Data | Time | Data interpretation | Data sources |
| --- | --- | --- | --- |
| Building data | 2019 | Vector data | http://ditu.amap.com |
| POI | 2019 | Point data | http://ditu.amap.com |
| LUUC | 2018 | Raster data  Landsat 8-OLI (Resolution 30 m) | http://www.resdc.cn/ |
| Remote sensing | 2019-08-12 | Landsat 8-OLI (Resolution 30 m) | glovis.usgs.gov |
| DEM | 2009 | SＲTM DEM (Resolution 30 m) | http://www.gscloud.cn/ |

## 3. Methods and factor selection

### 3.1Mono-window algorithm

LST is one of the important parameters in the study of surface energy balance. The commonly used methods of remote sensing inversion of LST mainly include the radiative transfer equation method, single window algorithm, single channel algorithm, and split window algorithm. Qin et al. (2001) analyzed atmospheric water vapor content using the mono-window algorithm and found a significant negative correlation between atmospheric transmittance and the inversion error of LST (Song et al., 2015; Sun et al., 2018). Nanjing has many water bodies, and summer is mostly hot and humid. As the area features low atmospheric permeability and high accuracy of LST inversion, Landsat TM 6 band and Landsat 8 TIRS 10 were selected, and a mono-window algorithm was used to invert LST. The inversion formula can be expressed as follows:

$T_{S}=\left\{ a_{6}\left( 1-C_{6}-D_{6} \right)+\left[ b_{6}\left( 1-C_{6}-D_{6} \right)+C_{6}+D_{6} \right]T_{6}-D_{6}T_{a} \right\}/C_{6}$	(1)

$C_{6}=\varepsilon_{6}\tau_{6}$ (2)

$D_{6}=(1-\varepsilon_{6})\left[1+\left(1-\varepsilon_{6}\right)\tau_{6}\right]$ (3)

where *T_S_* is surface inversion temperature (K); *a_6_* and *b_6_* are constants; *T_6_* is luminance temperature (K); *T_a_* is the average temperature of the atmosphere (K); and *C_6_* and *D_6_* are intermediate variables, which can be obtained from *ε_6_* (surface specific emissivity) and *τ_6_* (atmospheric transmittance in the thermal infrared band). According to existing studies, *a_6_*=-67.355351, *b_6_*=0.458606.

### 3.2 MGWR model

Compared with the classic geographically weighted regression (GWR) model, the kernel function and bandwidth selection of MGWR continue the selection criteria in the classic GWR, but the MGWR model adds spatially stable variables, and each regression coefficient *β_bwj_* is based on local regression. Moreover, each bandwidth is different. The calculation formula is as follows:

$yi=\sum_{j=1}^{k} \beta_{bwj}\left( u_{i},v_{i} \right)x_{ij}+\varepsilon_{i}$	(4)

where *bwj* is the bandwidth of the regression coefficient of the *j-th* variable, (*u_i_*, *v_i_*) represents the coordinates of the *i-th* local point in geographic space, *x_ij_* is the influencing factor, and *ε_i_* is the random error term.

### 3.3 Selection of impact factors

According to the principle of surface heat radiation and thermodynamic characteristics, LST is affected by thermal channels and near-surface gas LST. In low-altitude areas such as Nanjing, changes in surface radiation caused by topography, impervious surfaces, human Activity significantly affect the regional surface radiation. Referring to previous studies (Brunsdon et al., 1996; Li et al., 2017; Liu et al., 2019; Wu et al., 2020; Yan et al., 2019; Zhou and Tian, 2020), combined with the actual situation in the study area, and considering issues such as data availability, this study selected the factors listed in Table 3.

Table 2 Descriptions of major explanatory variables

| Variable name | Unit | Description |
| --- | --- | --- |
| *Intercept* | ℃ | Model intercept term, inversion of location factors |
| *Altitude* | m | Altitude of Nanjing |
| *Urban Green Space* | m^2^ | Urban green space and park distribution in Nanjing |
| *Road* | Classification | Distribution of roads of grade three and above |
| *Building Height (BH)* | Classification | 1 is a low-rise building, 2 is a high-rise building, and 3 is a super high-rise building |
| *Building Density (BD)* | Classification | 1 is low density, 2 is medium density building, 3 is high density building |
| *POI* | - | Spatial distribution of industrial activities |

## 4. Results and analysis

### 4.1 Spatial pattern characteristics of LST

Using Landsat 8 images, the overall distribution of LST was inversed according to the single window algorithm (Fig. 2). The highest LST of Nanjing was 37.121 ℃, the lowest was 19.923 ℃, and the average was 28.525 ℃. According to an analysis of hot spots, excluding outliers and the spatial distribution of LST (Fig. 3), the lowest LST in Xuanwu District is 22.978 ℃, and 413 cold spot pixels were identified with a confidence level of 0.99, accounting for 0.074 of the whole regions; the highest LST was 31.570 ℃, and 2172 hot spot pixels were identified with a confidence level of 0.99, accounting for 0.391 of the whole regions. In Yuhuatai District, the lowest LST was 22.924 ℃, and 98 cold spot pixels were identified with a confidence level of 0.99, accounting for 0.030 of the whole regions; the highest LST was 34.608 ℃, and 331 hot spot pixels were identified with a confidence level of 0.99, accounting for 0.103 of the whole regions. In Qinhuai District, the highest LST was 31.744 ℃, the lowest was 24.506 ℃, and 24 cold spot pixels were identified with a confidence level of 0.90, accounting for 0.006 of the whole regions. In Gulou District, the lowest LST was 22.956 ℃, and 39 cold spots were identified with a confidence level of 0.99, accounting for 0.008 of the whole regions. The highest LST in Jianye District was 33.677 ℃ and the lowest was 22.887 ℃; 169 cold spot pixels were identified with a confidence level of 0.99, accounting for 0.047 of the whole regions. The lowest LST in Qixia District was 19.923 ℃, and 61 cold spot pixels were identified with a confidence level of 0.99, accounting for 0.030 of the whole regions; the highest LST was 37.121 ℃, and 838 hot spot pixels were identified with a confidence level of 0.95, accounting for 0.410 of the whole regions. In Pukou District, the lowest LST was 22.845 ℃, 481 cold spot pixels were identified with a confidence level of 0.99, accounting for 0.106 of the whole regions; the highest LST was 35.846 ℃, and 2771 hot spot pixels were identified with a confidence level of 0.95, accounting for 0.609 of the whole regions. The lowest LST in Luhe District was 20.666 ℃, and 927 cold spot pixels were identified with a confidence level of 0.99, accounting for 0.147 of the whole regions; the highest LST was 33.437 ℃, and 3452 hot spot pixels were identified with a confidence level of 0.99, accounting for 0.550 of the whole regions. In Lishui District, the lowest LST was 20.204 ℃, and 694 cold spot pixels were identified with a confidence level of 0.99, accounting for 0.131 of the whole regions; the highest LST was 33.871 ℃, and 3323 hot spot pixels were identified with confidence level of 0.95, accounting for 0.629 of the whole regions. In Jiangning District, the lowest LST was 21.724 ℃, and 761 cold spot pixels were identified with a confidence level of 0.99, accounting for 0.163 of the whole regions; the highest LST was 36.274 ℃, and 2056 hot spot pixels were identified with a confidence level of 0.99, accounting for 0.441 of the whole regions. In Gaochun District, the lowest LST was 21.104 ℃, and 417 cold spot pixels were identified with a confidence level of 0.99, accounting for 0.119 of the whole regions; the highest LST was 35.072 ℃, and 1681 hot spot pixels were identified with a confidence level of 0.99, accounting for 0.48 of the whole regions.

Table 3 Statistics of LST in each district

| District | Minimum Temperature | Maximum Temperature | Cold spot | Proportion | Hot spot | Proportion |
| --- | --- | --- | --- | --- | --- | --- |
| Xuanwu | 22.978 | 31.570 | 413*** | 0.074 | 2172*** | 0.391 |
| Yuhuatai | 22.924 | 34.608 | 98*** | 0.030 | 331*** | 0.103 |
| Qinhuai | 24.506 | 31.744 | 24* | 0.006 | Not significant | Not significant |
| Gulou | 22.856 | 30.141 | 39*** | 0.008 | Not significant | Not significant |
| Jianye | 22.887 | 33.677 | 169*** | 0.047 | Not significant | Not significant |
| Qixia | 19.923 | 37.121 | 61*** | 0.030 | 838** | 0.410 |
| Pukou | 22.845 | 35.846 | 481*** | 0.106 | 2771** | 0.609 |
| Luhe | 20.666 | 33.437 | 927*** | 0.147 | 3452 | 0.550 |
| Lishui | 20.204 | 33.871 | 694*** | 0.131 | 3323** | 0.659 |
| Jiangning | 21.724 | 36.274 | 761*** | 0.163 | 2056*** | 0.441 |
| Gaochun | 21.104 | 35.072 | 417*** | 0.119 | 1681*** | 0.48 |

***indicates a confidence level of 0.99 , ** indicates a confidence level of 0.95, * indicates a confidence level of 0.90.

Overall, hotspots in Nanjing are concentrated mainly in Xuanwu District, Qixia District, Pukou District, Lishui District, Jiangning District, and Gaochun District. The highest value of LST was 37.12 ℃ in Qixia District, over an area located near the Lingang Industrial Concentration Zone in Qixia District and Nanjing Economic and Technological Development Zone. The area along the river between the two zones is largely covered by industrial land and residential land, with little vegetation. At the same time, the lowest value of LST in Nanjing was also observed in Qixia District, specifically in the Qixia Mountain. The area lies 268 m above sea level. The land use type is woodland, and the vegetation coverage is relatively high, with less interference from human activities. The distribution characteristics of LST hotspots in other areas are related to the cluster distribution of urban land, rural residential areas and other construction land, and the scattered distribution of waters and forests and grasslands, and are strongly affected by human activities.

Fig.2 Spatial distribution of LST in Nanjing

Fig.3 Hot spot analysis of LST

### *4.2 Distribution of impact factors*

After cleaning, calibrating, deleting anomalies, and other processing of building data, a total of 164,581 building areas were obtained (Fig. 4a). Low-rise, high-rise, and super high-rise buildings accounted for 64.20%, 32.84%, and 2.96%, respectively. Low-, medium-, and high-density buildings accounted for 57.89%, 26.60%, and 15.50%, respectively. The proportion of building area in each district is as follows: Qinhuai District (29.23%) > Xuanwu District (15.17%) > Yuhuatai District (11.16%) > Jianye District (9.91%) > Jiangning District (9.91%) > Pukou District (9.65%) > Qixia District (8.75%) > Luhe District (4.02%) > Lishui District (1.751%) > Gaochun District (0.420%) > Gulou District (0.03%).

Regarding land occupied by buildings in terms of BH (Table 4), Gaochun District has fewer building areas (blocks) at 692 blocks, mainly comprising low-rise buildings with a small number of high-rise buildings. Gulou District has a very high proportion of super high-rise buildings, with a very low proportion of low-rise buildings and a moderate proportion of high-rise civil buildings. Low-rise buildings are mainly distributed in Jiangning District, Yuhuatai District, Qinhuai District, Pukou District, and Xuanwu District. Lishui District and Qixia District mainly comprise low-rise and high-rise buildings, with a relatively low proportion of high-rise buildings. Jianye District is dominated by low-rise buildings, with high-rise buildings accounting for 38.24%, and the proportion of super high-rise buildings is relatively low. The main building types in Luhe District are low-rise and high-rise buildings, with a low distribution of super high-rise buildings.

Regarding the proportion of land occupied by buildings in terms of BD (Table 4), Gaochun District is dominated by low-density buildings, with a very low proportion of medium- and high-density buildings. The proportion of buildings is relatively low in Gulou District, with high-density buildings accounting for 89.09%, and low- and medium-density buildings accounting for 10.91%. Pukou District and Xuanwu District are dominated by low-density buildings, with no medium-density buildings; Yuhuatai District and Qinhuai District mainly include low-density buildings, with some moderate and high-density buildings; Lishui District and Jiangning District mainly include low-density buildings and medium-density buildings. Jianye District has a relatively high proportion of low-density buildings, and a moderate proportion of medium- and high-density buildings. Qixia District is dominated by low- and medium-density buildings. Moreover, the proportions of the two are similar, and the distribution of high-density buildings is relatively small. Luhe District has a relatively high proportion of medium-density buildings, and moderate proportions of low- and high-density buildings.

Table 4 Statistics of buildings in each district

| District | Building  area  (piece) | Proportion of various building heights (BH） | | | Proportion of building density (BD) | | |
| --- | --- | --- | --- | --- | --- | --- | --- |
|  |  | Low-rise buildings | High-rise buildings | Super high-rise buildings | Low-density buildings | Medium-density buildings | High-density buildings |
| Gaochun | 692 | 96.53 % | 3.47 % | 0.00 % | 96.24 % | 3.03 % | 0.72 % % |
| Lishui | 2881 | 50.57 % | 46.27 % | 3.16 % | 47.66 % | 33.32 % | 19.02 |
| Jiangning | 16312 | 62.31 % | 35.53 % | 2.16 % | 49.66 % | 35.78 % | 14.57 % |
| Yuhuatai | 18362 | 65.60 % | 31.19 % | 3.21 % | 60.11 % | 22.06 % | 17.84 % |
| Jianye | 16317 | 55.28 % | 38.24 % | 6.48 % | 50.59 % | 24.03 % | 25.39 % |
| Qinhuai | 48104 | 67.48 % | 30.23 % | 2.29 % | 62.15 % | 22.19 % | 15.66 % |
| Gulou | 55 | 7.27 % | 21.82 % | 70.91 % | 5.46 % | 5.45 % | 89.09 % |
| Xuanwu | 24962 | 68.41 % | 30.30 % | 1.29 % | 61.57 % | 26.22 % | 12.21 % |
| Qixia | 14398 | 47.88 % | 46.07 % | 6.06 % | 41.14 % | 40.66 % | 18.20 % |
| Pukou | 15884 | 76.11 % | 21.08 % | 2.81 % | 72.07 % | 18.30 % | 9.64 % |
| Luhe | 6614 | 38.25 % | 52.60 % | 9.15 % | 30.72 % | 44.80 % | 24.48 % |

Fig.4 Spatial distribution of impact factors

POI represents the distribution of urban physical facilities. After data crawling and cleaning, a total of 238423 POI were obtained, with a bandwidth of 0.6 km. The core density of POI in Nanjing was 2.18–996.91/km^2^, showing a spatial distribution of "low around and high in the center" (Fig. 4b). The distribution of POI and nuclear density in each region are shown in Table 5. The overall number of POI in Jiangning District, Pukou District, and Luhe District was higher than that in other districts, and the overall number of POI was the lowest in Gaochun district. With an extremely low value, the regional nuclear density was the lowest in Yuhuatai District, whereas it was the highest in Qinhuai District. The spatial clustering distribution of POI in each region exhibited different trends. Qixia District and Luhe District corresponded to the distribution of "upper low high", with percentages of points between the lowest value and standard deviation of 61.16% and 53.49%. Jiangning District and Yuhuatai District showed a distribution trend of "low in the lower part and high in the upper part", with percentages of points between the lowest value and standard deviation of 61.66% and 52.73%, respectively. Pukou District and Jianye District showed a distribution trend of "left low and right high", with percentages of 56.915% and 56.64%, respectively, for points between the minimum value and standard deviation. Xuanwu District and Qinling District showed percentages of 56.915% and 56.64%, respectively. Huaihe District exhibited a distribution trend of "right low, left high" with percentages of 51.86% and 43.01%. Gaochun District and Lishui District showed distribution trends of "low edge, high middle", with percentages of 52.66% and 51.64%. The overall distribution of POI nuclear density in Gulou District was uniform.

Table 5 POI statistics of each district

| District | POI (piece) | Proportion (%) | Density (pcs/km^2^) | Standard deviation |
| --- | --- | --- | --- | --- |
| Gaochun | 10517 | 4.411 | 2.852–727.334 | 65.092 |
| Lishui | 14788 | 6.202 | 2.938–749.145 | 62.990 |
| Jiangning | 59429 | 24.926 | 3.046–776.841 | 94.372 |
| Yuhuatai | 16329 | 6.849 | 2.183–556.779 | 112.759 |
| Jianye | 10592 | 4.443 | 2.677–682.616 | 149.710 |
| Qinhuai | 15374 | 6.448 | 3.909–996.906 | 193.122 |
| Gulou | 18938 | 7.943 | 3.540–902.626 | 183.731 |
| Xuanwu | 14226 | 5.967 | 3.085–786.590 | 145.226 |
| Qixia | 23998 | 10.065 | 2.377–606.095 | 91.722 |
| Pukou | 28579 | 11.987 | 2.841–724.402 | 92.142 |
| Luhe | 25653 | 10.759 | 2.332–594.628 | 72.375 |
| Total | 238423 | 100 |  | |

After checking the topology of road network data, they were calibrated based on Google Earth Pro2019. Referring to existing research and the actual situation of the research area, highways, urban traffic arterial roads, and urban branch roads were reserved (Fig. 4c), setting a 1 km × 1 km grid, and the road network density (road network density = total road length km/grid area km^2^) was calculated. The results are shown in Table 6, for which abnormal values were removed. The higher the road network density, the stronger the regional road accessibility, promoting travel among urban residents. The higher the connectivity, the more convenient the regional traffic; conversely, the lower the density of the road network, the less the distribution of arterial roads and highways in the area, which greatly reduces road accessibility and decreases the convenience of residents to travel. Combining Figs. 1 and 2, the road network in Nanjing shows uneven development. Areas with high levels of road network density are mainly distributed in the central area, including the districts of Jianye, Gulou, and Yuhuatai, which may be clustered with buildings. It is related to the dense distribution of entertainment venues such as cultural centers. Areas with low levels of road network density are mainly distributed in the south, northeast, and northwest regions, such as the districts of Gaochun, Qixia, Luhe, and Pukou, which may have large areas of water and forests. The distribution of grassland and farmland is related to the scattered distribution of rural settlements.

Table 6 Road statistics of each district

| District | Road network density (km/km^2^) | Standard deviation (km/km^2^) |
| --- | --- | --- |
| Gaochun | 0.011–15.645 | 2.933 |
| Lishui | 0.009–15.516 | 3.436 |
| Jiangning | 0.004–15.573 | 3.853 |
| Yuhuatai | 0.037–18.111 | 5.377 |
| Jianye | 0.068–16.816 | 6.174 |
| Qinhuai | 1.109–14.263 | 4.910 |
| Gulou | 0.143–15.805 | 5.647 |
| Xuanwu | 0.110–14.534 | 5.163 |
| Qixia | 0.009–12.112 | 4.431 |
| Pukou | 0.004–14.918 | 3.199 |
| Luhe | 0.002–12.878 | 3.371 |

In terms of altitude and urban green space, Nanjing is in a low altitude area, dominated by lacustrine plains and valley bottoms, with a small amount (39.2% of the total area) of undulating mountains and hills, plains, depressions, rivers, and lakes. The Ningzhen Mountains and Jiangbei Laoshan straddles the central part of the city, and the south is a geomorphologically complex area composed of topographical units, such as low mountains, valley plains, and rivers. As mountainous areas, the highest altitudes of Xuanwu, Pukou, and Jiangning districts are higher than those of other regions (Fig. 4e). The density of urban green space (total area of urban gardens and green space/total area of urban land) is shown in Fig. 4d. The urban green space in Nanjing shows a widely variable spatial distribution, mainly concentrated in Xuanwu Lake, Zijin Mountain, and ancient city walls in the city center. Overall, in addition to urban green spaces near the "Central Park", they are concentrated in the Riverside Scenic Belt, Qinhuai River Scenic Belt, and Pukou Central Park.

### 4.3 Impact factor analysis

To quantitatively analyze the spatial distribution characteristics of LST and its factors in Nanjing, altitude, urban green space, slope, aspect, BD, BH, and POI were analyzed. A correlation test of the variables was conducted, and the results showed that the *Moran’s* between slope and aspect is less than 0.2, with a small spatial correlation. As this index failed the *Moran’s* test, it was not included in the global variable. The remaining indexes all showed values greater than 0.7, with some at the 1% level, showing significant spatial positive correlation. Accordingly, they were included in the local variables for calculation.

Table 4 shows that the goodness of fit (R^2^) of MGWR is slightly higher than that of the precision GWR model, and the value of the corrected Akaike information criterion (AICc) is also lower than that of the classic GWR model. Therefore, MGWR can be assessed to have higher performance than the classic GWR. Comparing the residual sum of squares, the value of MGWR was also smaller than that of GWR. Moreover, MGWR requires fewer parameters to obtain a regression result closer to the true value.

The spatial distribution of each influencing factor is shown in Fig. 4. The MGWR analysis results show that the spatial distribution of each influencing factor and LST have significant similarities and differences. LST decreased with increasing altitude, urban green space, and BH, whereas it increased with increasing values of BD and POI. The goodness of fit between POI and LST was the highest, reaching 0.96, followed by that between BD and LST (0.95) and between BH and LST (0.94). The values of goodness of fit between altitude, urban green space and LST were good (0.73, 0.61, respectively), and that between aspect and LST was average (0.37). These results further show that the natural environment is the basic factor affecting the spatial distribution of LST, and changes in the surface environment caused by human activities have a particularly significant impact on urban LST. Industrial facilities and business districts, such as Xinjiekou business district in Qinhuai District, Taipingmen business district in Xuanwu District, and Shuiximen business district in Jianye District, are concentrated in Xuanwu District, Qinhuai District, Gulou District, Jianye District, and other downtown areas, which also account for the main low-rise buildings and super-high buildings in Nanjing. At the same time, a large number of buildings are concentrated in the central area, which further increases the flow of people. The concentrated POI distribution has a significant positive correlation with the spatial distribution of LST.

Table 4 Model index of GWR and MGWR

| Model | MGWR | GWR |
| --- | --- | --- |
| R^2^ | 0.928 | 0.883 |
| AIC_C_ | 1303 | 1186 |
| Adj. R^2^ | 0.902 | 0.875 |
| Residual sum of squares | 1323 | 1977 |

Table 5 Statistical description of MGWR coefficients

| Variable | Min | Max | Median | Std | Mean |
| --- | --- | --- | --- | --- | --- |
| *Intercept* | -0.49 | 1.46 | 0.485 | 0.975 | 0.485 |
| *Arterial Road* | -0.42 | 1.41 | 0.495 | 0.915 | 0.495 |
| *Secondary Road* | -0.34 | 1.53 | 0.595 | 0.935 | 0.595 |
| *Access Road* | 0.76 | 1.97 | 1.365 | 0.605 | 1.365 |
| *BuildingHeight.1* | -0.14 | 0.21 | 0.035 | 0.175 | 0.035 |
| *BuildingHeight.2* | 0.45 | 3.01 | 1.73 | 1.28 | 1.73 |
| *BuildingHeight.3* | -0.6 | 3.76 | 1.58 | 2.18 | 1.58 |
| *Urban Green Space* | -1.42 | 0.26 | -0.58 | 0.84 | -0.58 |
| *Compact Highrise* | -0.43 | 0.67 | 0.14 | 0.55 | 0.12 |
| *Open Midrise* | -0.52 | 3.68 | 1.55 | 2.1 | 1.58 |
| *Lightweight Rise* | 0.29 | 1.2 | 0.741 | 0.455 | 0.745 |
| *POI* | -0.72 | 1.6 | 0.47 | 1.16 | 0.44 |
| *DEM* | -2.5 | -0.25 | -1.381 | 1.125 | -1.375 |

The statistical description of each coefficient of MGWR is shown in Table 5. The *Intercept* represents the positive influence of location factors on LST. The value of *Intercept* was between -0.49 and 1.46, the average value was 0.485, and the standard deviation was 0.975, indicating that under the same natural conditions, the influencing factors would change the LST of Nanjing by -0.49–1.46 ℃, with an average change of 0.485 ℃. The influence of location factors on LST widely varies.

Urban roads have a significant positive impact on LST. Herein, roads in Nanjing are divided into three levels according to the classification standards of urban roads: *Arterial Road*, *Secondary Road*, and *Access Road*. *Arterial Road* ranges from -0.42 to 1.41, with an average value of 0.495, *Secondary Road* ranges from -0.34 to 1.53, with an average value of 0.595, and *Access Road* ranges from 0.76 to 1.97, with an average value of 1.365. These results show that in urban roads, branch roads have a greater impact on LST. In other words, under the circumstance that the influence of other factors remains unchanged, the LST around a branch road (*Access Road*) would be approximately 1.365 °C higher than that in the surrounding areas. Therefore, the density of urban roads will also affect LST.

Referring to the literature (Liu and Shi, 2020), quantitative indicators of local climate zones, and the actual situation of Nanjing, the study area was divided into low-density low-rise building areas, medium-density high-rise building, areas and high-density super high-rise building area (Fig. 4-e), and the relationships between BH, BD, and LST were analyzed. Low-rise building areas (*BuildingHeight.1*) showed LST values ranging from -0.14 to 0.21, with an average value of 0.035. High-rise building areas (BuildingHeight.2) showed LST values ranging from 0.45 to 3.01, with an average value of 1.73. Super high-rise building areas (BuildingHeight.3) showed LST values ranging from -0.6 – 3.76, with an average value of 1.58. This further shows that there is a significant positive correlation between BH and LST. From the perspective of the absolute value of the coefficient, the area of middle-rise buildings has the greatest impact on LST. In terms of BD, the value of the impact of high-density high-rise buildings on LST ranged from -0.43 to 0.67, with an average value of 0.12 ℃. The value for medium-density high-rise building areas ranged from -0.52 to 3.68, with an average value of 1.58 ℃. The value for low-density low-rise buildings ranged from 0.29 to 1.2, with an average value of 0.745 ℃. The absolute value of the influence coefficient shows that high-rise buildings and medium-density mid- and high-rise buildings have the greatest influence on LST. Previous studies suggested that increases in BH will increase LST. In contrast, this study shows that increases in BH do not necessarily lead to an increase in LST. This can be attributed to increased shadow areas generated by high-rise buildings, leading to lower temperatures within a certain range (Meng et al., 2018).

Urban green space and LST in Nanjing exhibit a negative correlation, with an impact coefficient of -0.58°C, indicating that increases in urban green space will decrease LST. Under the condition that other factors remain unchanged, the LST of urban green space is lower than that of surrounding areas by 0.58 ℃. In contrast, POI has a positive correlation with LST. The higher the intensity of human activities, the higher the LST, but its impact coefficient is 0.44, which is moderate. The DEM coefficient ranged from -2.5 to -0.25. This implies that the LST of areas at higher altitudes is lower than that of areas at lower altitudes by 1.375 ℃ on average.

## 5 Conclusion

In this study, the MGWR model was used to determine the relationship between urban form and surface temperature for the first time. Combining POI data, building data, and remote sensing data of Nanjing, the spatial differentiation of urban form (two-dimensional landscape pattern and three-dimensional structure form) and LST and its influencing factors were analyzed. The following main conclusions can be drawn:

(1) The spatial differentiation of LST in Nanjing is significant, and the distribution of LST hotspots exhibits a distinct spatial consistency with the level of urban construction. The highest LSTs in Qixia District (37.121°C), Jiangning District (36.274°C), and Pukou (35.846°C) are higher than those in other areas, and the lowest LSTs in Qixia District (19.923°C), Lishui District (20.204°C), and Luhe District (20.666°C) are lower than those in other areas.

(2) Compared with classic GWR, MGWR supports the analysis of multiple influencing factors or variables at different scales and provides better fitting effect than GWR. In this study, some factors exhibited significant differences in their effects on LST. Except for the aspect and slope, the other influencing factors all showed significant spatial correlation with LST. In terms of the two-dimensional landscape pattern, the higher the altitude, the lower the LST; the higher the POI concentration, the higher the LST. In terms of the three-dimensional structure, BH and LST are positively correlated.

## 6. Discussion

The conclusions of this research and existing research results are mutually confirmed (Han et al., 2016; Yan et al., 2019). In plain areas with low altitude and gentle slopes, the development of human activities is less difficult, human activities are frequent, and construction land is concentrated. Areas with concentrated secondary and tertiary industries are more likely to have higher LST, whereas areas with mountains and agricultural land are likely to have lower LST due to several factors such as altitude and vegetation. It is noteworthy that, unlike the findings of previous studies, an obvious positive correlation was found between urban BH, BD, and LST. However, the LST of high-rise and super high-rise building areas was found to be lower than that of mid-rise building areas. This could be explained by the expansion of shadow areas generated by super high-rise buildings; similar phenomena were also observed between medium-density building areas and high-density building areas (Meng et al., 2018; Peng et al., 2021).

A city is a complex system where residents provide certain infrastructure and social relations. The urban thermal environment is the result of a combination of human activities and other factors under certain natural conditions (Zhao et al.,2020; Chen et al.,2019). Due to the problem of data acquisition, the research on the relationship between surface temperature and its influence at home and abroad mostly adopts top-down methods, to a certain extent Ignore the influence of human activities and urban form.

Based on the existing research, this paper determines the impact of different human activities on the urban thermal environment, and further proves that urban green space can help alleviate the urban heat island effect. (Zhou et al.,2018; Zhao et al.,2020). The process of urbanization has led to overpopulation and excessive industrial concentration, causes a change in the nature of heat exchange at the bottom, and aggravating the formation and development of the urban heat island effect, which requires the attention of urban planning agencies. In addition, based on the results of this article, strategies to reduce heat stress by addressing the urban heat island effect, (e.g., control the scale of urban built-up areas, optimize urban spatial structure, increase urban green areas, alleviate urban population concentration and other measures). We should also cooperate with commercial real estate developers to control the height and density of new buildings, optimize the design of future urban parks, increase the construction of urban ventilation corridors and green spaces, and further alleviate the urban heat island effect (Liu et al.,2020).

## 7 Advantages and Limitations

### 7.1 Advantages

Based on research of urban thermal environment effect for a single data source influencing factors, and the problem of analysis method of single with higher level of urbanization in Nanjing as the research object, combined with digital elevation data urban building land use type data and POI data set multi-source spatial information data, comprehensive utilization of quantitative inversion and MGWR analysis methods, such as, on the basis of comprehensive study and the urban thermal environment effect and influence factors, further enrich the research of urban thermal environment research perspective Studies have proved that Geography Weighted Regression(GWR) has been broadly used in various fields to model spatially non-stationary relationships(Liu et al.,2018;Yang et al.,2020).Multi-scale Geographically Weighted Regression(MGWR) is a recent advancement to the classic GWR model. Compared with the traditional GWR model, The MGWR model has advantages in acquiring the ability of different scales (Jin et al.,2021). The MGWR model can effectively analyze the multi-scale relationship between the urban thermal environment and its influencing factors, and has a positive effect on urban dynamic development and urban thermal environment management.
7.2 Limitations

This study integrated POI data, building data, urban road data sets and other data to analyze the factors affecting the urban thermal environment. However, it should also be noted that POI data represents the geographic information and utilization characteristics of various facilities, and can only represent a certain Whether the utilization characteristics of each time node can represent the historical utilization characteristics and intensity still needs to be studied. Secondly, existing researches mostly focus on the status quo of urban thermal environment, and there is still a lack of in-depth research on the prediction and analysis of urban thermal environment evolution trends, the construction of a heat island effect warning mechanism, and urban space optimization strategies.

The MGWR model facilitated multi-factor analysis of LST. However, due to issues such as data availability and collinearity, the application of the MGWR model has certain limitations. The urban landscape is a complex dynamic system composed of infrastructure, human activities, and social connections. Changes in urban surface temperature need to be observed from a more micro perspective (Cao et al., 2021; Li et al., 2020). Urban ground monitoring data have not been fully disclosed, which limits the study. In addition, street view data were used in the study of the urban thermal environment, and the number of street scenes in this area requires further investigation (Zhang et al., 2019). In the future, the interaction between different influencing factors should be considered, and the influencing factors of LST should be analyzed in more detail to provide a more comprehensive perspective for urban or regional environmental governance and planning.

## Credit authorship contribution statement

Shusheng Yin: Methodology, Data curation, Writing Original draft preparation.

Jiatong Liu: Methodology, Visualization, Editing.

Zenglin Han: Conceptualization, Methodology, Writing- Reviewing and Editing.

## Declaration of competing interest

The manuscript has not been published before and is not being considered for publication elsewhere. All authors have contributed to the creation of this manuscript for important intellectual content and read and approved the final manuscript. We declare there is no conflict of interest.

## Acknowledgements

This material is partially based upon work supported by the National Science Foundation under Grant NO. 41976206. Any opinions, findings, and conclusions or recommendations expressed in this material are those of the author and do not necessarily reflect the views of the National Science Foundation.

## Reference

Alexander Buyantuyev, Jianguo Wu. Urban heat islands and landscape heterogeneity: linking spatiotemporal variations in surface temperatures to land-cover and socioeconomic patterns [J]. Landscape Ecology, 2010,25:17–33. DOI 10.1007/s10980-009-9402-4.

Bin Zhou, Diego Rybski, Jürgen P. Kropp. The role of city size and urban form in the surface urban heat island [J]. Science Reports, 2017,(07):1-9. DOI.10.1038/s41598-017-04242-2.

Blessing Bolarinwa Fabeku, Ifeoluwa Adebowale Balogun, Suleiman Abdul-Azeez Adegboyega, Orimoloye Ipoola Faleyimu. Spatio-Temporal Variability in Land Surface Temperature and Its Relationship with Vegetation Types over Ibadan, South-Western Nigeria [J]. Atmospheric and Climate Sciences, 2018,8(3):319-336.DOI:10.4236/acs.2018.83021.

Brunsdon C, Fontheringham A S, Charlton M E. Geographically Weighted Regression:A Method for Explor ing Spatial Nonstationarity [J]. Geographically Analysis, 1996,28(4):281-298. DOI:10.1111/j.1538-4632.1996.tb00936.x.

Cao Jie, Zhou Weiqi, Zheng Zhong, Ren Tian, Wang Weimin. Within-city spatial and temporal heterogeneity of air temperature and its relationship with land surface temperature [J]. Landscape and Urban Planning, 2021,206. DOI: 10.1016/j.landurbplan.2020.103979.

Cheng He, Liang Ma, Liguo Zhou, HaiDong Kan, Yan Zhang, WeiChun Ma, Bin Chen. Exploring the mechanisms of heat wave vulnerability at the urban scale based on the application of big data and artificial societies [J]. Environment International, 2019,127. 573-583.DOI:10.1016/j.envint.2019.01.057.

Dechao Chen, Xinliang Xu, Zongyao Sun, Luo Liu, Zhi Qiao, Tai Huang. Assessment of Urban Heat Risk in Mountain Environments: A Case Study of Chongqing Metropolitan Area, China[J]. Sustainability,2019,12(1). DOI:10.3390/su12010309.

Decheng Zhou, Jingfeng Xiao, Stefania Bonafoni, Christian Berger, Kaveh Deilami, Yuyu Zhou, Steve Frolking,R ui Yao, Zhi Qiao ,José A. Sobrino. Satellite Remote Sensing of Surface Urban Heat Islands: Progress, Challenges, and Perspectives[J]. Remote Sensing,2018,11(1). DOI:10.3390/rs11010048.

Feng Lei, Zhang Kaiyi, Gao Jingjing, Dai Zhixiu. Study of road surface temperature by using the land surface temperature retrieval products from meteorological satellite data [J]. Science and Technology Review, 2019,37(20):49-64.

Gu Guofeng, Li Qiao, Zhou Yinan. Evolution of Economic Growth Spatial Correlation Network Structure of Urban Agglomeration in Northeast China [J]. Area Research and Development, 2020,39(02):14-19.DOI:10.3969 /j. issn.1003-2363.2020.02 .003.

Gu Hengyu, Qin Xiaoling, Shen Tiyan. Spatial variation of migrant population's return intention and its determinants in China's prefecture and provincial level cities [J]. Geographical Research, 2019,38(08):1877-1890. DOI: 10.11821/dlyj020180473.

Han G F, Ye L, Sun Z W. Influence of aspect on land surface temperature in mountainous city: a case study in central area of Chongqing City. Acta Ecologica Sinica, 2014，34(14):4017-4024. DOI: 10.5846/stxb201211271679.

Han Guifeng, Cai Zhi, Xie Yusi, Zeng Wei. Correlation between urban construction and urban heat island: A case study in Kaizhou District, Chongqing [J]. Journal of Civil and Environmental Engineering, 2016,38(05):138-147. DOI: 10.11835/j.issn.1674-4764.2016.05.018.

Han Xiuzhen, Li Sanmei, Dou Fangli. Study of obtaining high resolution near-surface atmosphere temperature by using the land surface temperature from meteorological satellite data[J]. Acta Meteorologica Sinica, 2012,70(05):1107-1118.

Hooker Josh, Duveiller Gregory, Cescatti Alessandro. A global dataset of air temperature derived from satellite remote sensing and weather stations [J]. Scientific Data, 2018,5. DOI:10.1038/sdata.2018.246.

Hu Zhaolin, Dai Hui, Hou Fei, Li Erzhu. Spatio-temporal change of urban-rural vegetation phenology and its response to land surface temperature in Northeast China. Acta Ecologica Sinica, 2020, 40(12):4137-4145. DOI:10.5846/stxb201812252806.

Jian Peng, Yuzhuo Dan, Ruilin Qiao, Yanxu Liu, Jianquan Dong, Jiansheng Wu. How to quantify the cooling effect of urban parks? Linking maximum and accumulation perspectives [J]. Remote Sensing of Environment, 2021,252:112-135. DOI.: 10.1016/j.rse.2020.112135.

Jin Lina, Li Xiongfei, Du Mengmeng, et al． Analysis on the Characteristics of Urban Heat Island in Xi’an Based on FY-3 Satellite Data [J]. Meteorological and Environmental Sciences, 2019,42( 4):74－82. DOI: 10.16765 /j.cnki.1673－7148. 2019.04.011

[Jinlong Zang](https://xueshu.baidu.com/s?wd=author%3A%28Jinlong%20%20%20%20Zang%29%20&tn=SE_baiduxueshu_c1gjeupa&ie=utf-8&sc_f_para=sc_hilight%3Dperson)，[Qiaozhen Guo](https://xueshu.baidu.com/s?wd=author%3A%28Qiaozhen%20%20%20%20Guo%29%20&tn=SE_baiduxueshu_c1gjeupa&ie=utf-8&sc_f_para=sc_hilight%3Dperson)，[Xiaoxu Wu](https://xueshu.baidu.com/s?wd=author%3A%28Xiaoxu%20%20%20%20Wu%29%20&tn=SE_baiduxueshu_c1gjeupa&ie=utf-8&sc_f_para=sc_hilight%3Dperson)，[Xiao Sang](https://xueshu.baidu.com/s?wd=author%3A%28Xiao%20%20%20%20Sang%29%20&tn=SE_baiduxueshu_c1gjeupa&ie=utf-8&sc_f_para=sc_hilight%3Dperson)，[Huanhuan Wu](https://xueshu.baidu.com/s?wd=author%3A%28Huanhuan%20%20%20%20Wu%29%20&tn=SE_baiduxueshu_c1gjeupa&ie=utf-8&sc_f_para=sc_hilight%3Dperson)，[Yue Qiao](https://xueshu.baidu.com/s?wd=author%3A%28Yue%20%20%20%20Qiao%29%20&tn=SE_baiduxueshu_c1gjeupa&ie=utf-8&sc_f_para=sc_hilight%3Dperson). Integrated evaluation on multi-scale land surface temperature grading and bio-temperature suitability—a case study in Tianjin China[J]. International Journal of Remote Sensing,2021,42(1).

Jiyao Zhao, Le Yu, Yidi Xu, Xuecao Li, Yuyu Zhou, Dailiang Peng, Han Liu, Xiaomeng Huang, Zheng Zhou, Dong Wang, Chao Ren, Peng Gong. Exploring difference in land surface temperature between the city centers and urban expansion areas of China’s major cities[J]. International Journal of Remote Sensing,2020,41(23). DOI:[10.1080/01431161.2020.1797216](https://schlr.cnki.net/Detail/DOI/STJDLAST/STJD706AA602FF74C4A3F760578AA9EFEC5D).

Li Bin, Wang Huimin, Qin Mingzhou, Zhang Pengyan. Comparative study on the correlations between NDVI, NDMI and LST [J]. Progress in Geography, 2017,36(05):585-596. DOI: 10.18306/dlkxjz.2017.05.006.

Li Dan, Tian Peipei, Luo Hongying, Hu Tiesong, Dong Bin, Cui Yuanlai, Khan Shahbaz, Luo Yufeng. Impacts of land use and land cover changes on regional climate in the Lhasa River basin, Tibetan Plateau [J]. Science of The Total Environment, 2020,742. DOI: 10.1016/j.scitotenv.2020.140570.

Li Hui, Gu Ronghua, Zhu Yulin. Effect of Land Transfer on the Economic Radiation of Urban Agglomerations in the Middle Reaches of the Yangtze River and Formation Mechanism [J]. Resources and Environment in the Yangtze Basin, 2020,29(01):35-43. DOI: [10.11870/cjlyzyyhj202001004](https://doi.org/10.11870/cjlyzyyhj202001004).

Liu Lu, Shen Guangrong, Wu Yu, ZhangZhou Yilin, Lu Shaoming. Characteristics of Land Use Transfer and Its Influence on Thermal Environment in County-level Urbanization [J]. Bulletin of Soil and Water Conservation, 2019,39(06):260-266. DOI:10.13961/j.cnki.stbctb.2019.06.038.

Liu Yansui. Research on the urban-rural integration and rural revitalization in the new era in China [J]. Acta Geographica Sinica, 2018,73(04):637-650. DOI:10.11821/dlxb201804004.

Liu Yaobin, Leng Qingsong. Urbanization, Population Agglomeration and Haze Changes: Based on Threshold Regression and Spatial Partition [J]. Ecological Economy, 2020,36(03):92-98.

Liu Ye, Xue Yongkang, Li Qian, Lettenmaier Dennis, Zhao Ping. Investigation of the Variability of Near‐Surface Temperature Anomaly and Its Causes Over the Tibetan Plateau[J]. Journal of Geophysical Research: Atmospheres,2020,125(19). DOI：10.1029/2020JD032800.

Liya Chao, Boyin Huang, Yang Yuanjian, Phil Jones, Jiayi Cheng, Yang, Qingxiang Li. A New Evaluation of the Role of Urbanization to Warming at Various Spatial Scales: Evidence From the Guangdong‐Hong Kong‐Macau Region, China[J]. Geophysical Research Letters,2020,47(20). DOI：

Mashhoodi Bardia, Stead Dominic, van Timmeren Arjan. Spatializing household energy consumption in the Netherlands: Socioeconomic, urban morphology, microclimate, land surface temperature and vegetation data [J]. Data in Brief, 2020,29. DOI:10.1016/j.dib.2020.105118.

Meng Qingyan, Sun Yunxiao, Zhang Jiahui, Chen Xu, Sun Zhenghui. Assessing Vertical Greenery Distribution and Spatial Allocation based on Multi-source Remote Sening:A Case Study of Székesfehérvár City, Hungary, [J]. Remote Sensing Technology and Application, 2018,33(02):370-376. DOI：10.11873/J.ISSN.1004-0323.2018.2.0370.

Milan Gavrilović,Milan Pjević,Mirko Borisov,Goran Marinković,Vladimir M. Petrović. Analysis of Climate Change in the Area of Vojvodina-Republic of Serbia and Possible Consequences[J]. Journal of Geographical Research,2020,02(02):11-19. DOI.[10.30564/jgr.v2i2.952](https://schlr.cnki.net/Detail/DOI/WWMERGEJLAST/SJYB3A7B6B943BC1FC6A2E8D97F21F0E5DDF).

Mingxing Chen, Shasha Guo, Maogui Hu, Xiaoping Zhang. The spatiotemporal evolution of population exposure to PM 2.5 within the Beijing-Tianjin-Hebei urban agglomeration, China [J]. Journal of Cleaner Production, 2020. DOI.org/10.1016/j.jclepro.2020.121708.

National Bureau of Statistics http://data.stats.gov.cn

Oke T R. Canyon geometry and the nocturnal urban heat island: comparison of scale model and field observations [J]. International Journal of Climatology, 1981,1(3):237-254.

Qin Zhihao, Zhang Minghua, Arnon Karnieli, Pedro Berliner. Mono-window Algorithm for Retrieving Land Surface Temperature from Landsat TM6 data [J]. Acta Geographica Sinica, 2001(04):456-466. DOI:10.1142/S0252959901000401.

Qiu Lisha, He Yi, Zhang Lifeng, Wang Wenhui, Tang Yuanwei.	Spatiotemporal variation characteristics and influence factors of MODIS LST in Qilian Mountains [J]. Arid Land Geography, 2020,43(03):721-737. DOI:10.12118/j.issn.1000-6060.2020.0319.

Ricardo Fonseca, María‐Paz Zorzano‐Mier, Armando Azua‐Bustos, Carlos González‐Silva, Javier Martín‐Torres. A surface temperature and moisture intercomparison study of the Weather Research and Forecasting model, in‐situ measurements and satellite observations over the Atacama Desert [J]. Quarterly Journal of the Royal Meteorological Society, 2019,145(722).

Scarano Mancini. Assessing the relationship between sky view factor and land surface temperature to the spatial resolution [J]. International Journal of Remote Sensing, 2017,38(23):6910-6929. DOI: 10.1080/01431161.2017.1368099

Shengjie Liu, Qian Shi. Local climate zone mapping as remote sensing scene classification using deep learning: A case study of metropolitan China [J]. ISPRS Journal of Photogrammetry and Remote Sensing, 2020,164:229-242 DOI.10.1016/j.isprsjprs.2020.04.008

Shi Xin, Zhou Maichun, Liu Zhenhua, Hu Yueming, Liu Yuan, Zhu Shuhua. Comparative Analysis on Three Land Surface Temperature Inversion Algorithm based on Landsat 8 Data over Sanheba Basin [J]. Remote Sensing Technology and Application, 2018,33(03):465-475. DOI:10.11873/j.isn.1004-0323.2018.3.0465.

Song Ting, Duan Zheng, Liu Junzhi, Shi Junzhe, Yan Fei, Sheng Shijie, Huang Jun, Wu Wei. Comparison of four algorithms to retrieve land surface temperature using Landsat 8 satellite [J]. Journal of Remote Sensing, 2015,19( 3) : 451-464. DOI: 10.11834 /jrs. 20154180.

Srivanit M, Kazunori H. The influence of urban morphology indicators on summer diurnal range of urban climate in Bangkok metropolitan area, Thailand [J]. International Journal of Civil & Environmental Engineering, 2011,11(5):34-46.

Stewart I D , Oke T R . Local Climate Zones for Urban Temperature Studies[J]. Bulletin of the American Meteorological Society, 2012, 93(12):1879-1900.DOI:10.1175/BAMS-D-11-00019.1

Stewart I D. A systematic review and scientific critique of methodology in modern urban heat island literature [J]. International Journal of Climatology, 2011,31(2):200-217. DOI.10.1002/joc.2141.

Tang Chengli, Chen Weiyang, Wu Jiamin Zhou Guohua, Wang Meixia, Guo Xiashuang. Spatial distribution and industrial agglomeration characteristics of development zones in the Yangtze River Economic Belt. Scientia Geographica Sinica, 2020,40(4):657-664. doi:10.13249/j.cnki.sgs.2020.04.018.

Wan K W, Tsang C L, Lam J C. Sensitivity analysis of building energy use in different climates [J]. IFAC Proceedings Volumes, 2010, 43(1):58-62.DOI. 10.3182/20100329-3-PT-3006.00013

Wang Hui, Wang Qi. The Pollution Emission and Urbanization in China：Based on Input-output Analysis [J]. Chinese Journal of Population Science, 2011(05):57-66+111-112.

Wu Qiong, Gong Jian, Yang Jianxin. Multi-scale Anaysis of Main Factors of Summer Thermal Field in Wuhan Based on POI Data [J]. Resources and Environment in the Yangtze Basin, 2020,29(01):200-210. DOI:10.11870/cjlyzyyhj202001018.

Xiaoni Wang, Catherine Prigent. Comparisons of Diurnal Variations of Land Surface Temperatures from Numerical Weather Prediction Analyses, Infrared Satellite Estimates and In Situ Measurements [J]. Remote Sensing, 2020,12(3).

Xue Yayong, Liang Haibin, Zhang Yuan, Wang Xiaofeng. Spatial and Temporal Variations of Land Surface Temperature of the Loess Plateau [J]. Earth and Environment, 2017,45(05):500-507. DOI: 10.14050 /j.cnki.1672-9250.2017.05.002

Yan Guanghua, Su Junru, Guan Dunyi. The Impact of Urban Architectural Vertical Characteristics on Urban Thermal Environment in Zhongshan District, Dalian [J]. Scientia Geographica Sinica, 2019,39(1): 125-130. DOI: 10.13249/j.cnki.sgs.2019.01.014.

Yang Chen, Zhan Q, Gao Sihang, Liu Huimin. Characterizing the spatial and temporal variation of the land surface temperature hotspots in Wuhan from a local scale[J]. Geo-spatial Information Science,2020,23(4). DOI: [10.1080/10095020.2020.1834882](https://www.researchgate.net/deref/http%3A%2F%2Fdx.doi.org%2F10.1080%2F10095020.2020.1834882).

Yanxu Liu, Jian Peng, Yanglin Wang. Efficiency of landscape metrics characterizing urban land surface temperature[J]. Landscape and Urban Planning,2018,180. DOI:10.1016/j.landurbplan.2018.08.006.

Yao Yuan, Chen Xi, Qian Jin. Research progress on the thermal environment of the urban surfaces．Acta Ecologica Sinica, 2018,38( 3):1134-1147. DOI: 10.5846 /stxb201611022233.

Zhang Hui, Yuan Fenghui, Wang anzhi, Guan Dexin, Dai Guanhua, Wu Jiabing. Variation characteristics of NDVI and its response to climatic change in the growing season of Changbai Mountain Nature Reserve during 2001 and 2018 [J]. Chinese Journal of Applied Ecology, 2020,31(04):1213-1222. DOI:10.13287/j.1001-9332.202004.020.

Zhang Yujia, Middel Ariane, Turner B L. Evaluating the effect of 3D urban form on neighborhood land surface temperature using Google Street View and geographically weighted regression [J]. Landscape Ecology, 2019. DOI: 10.1007/s10980-019-00794-y.

Zhao Zi-qi, Li Li-guang, Wang Hongbo, et al. Study on the relationships between land use and land surface temperature in Shenyang urban districts [J]. Journal of Meteorology and Environment, 2016, 32(6):102－108. DOI: 10.3969 /j.issn.1673-503X.2016.06.013.

Zhou Weiqi, Tian Yunyu. Effects of urban three-dimensional morphology on thermal environment: a review [J]. Acta Ecologica Sinica, 2020,40( 2):416-427. DOI: 10.5846 /stxb201902250353.

1. [*http://data.stats.gov.cn*](http://data.stats.gov.cn)
