# Supplementary material for: Relationship between urban morphology and land surface temperature—A case study of Nanjing City
Source: PLoS One. 2022 Feb 9;17(2):e0260205. doi: 10.1371/journal.pone.0260205 (PMC8827472; doi:10.1371/journal.pone.0260205)
Supplement: Attachment — Submitted filename: Responses to Reviewers.docx [file pone.0260205.s002.docx]

Dear Roland Paile Bendaña

Thank you for sending the feedback. Overall, the manuscript has been extensively revised to address the concerns raised by the editor. The specific comments and changes are listed as follows in a point-to-point manner.

**Response to** **Editor Comments:**

1. *Thank you for uploading your figures as individual figure files. Before we can proceed, please still remove the images from the manuscript file, and upload an updated version of this doc.*

**Response #1:** We have removed the pictures in the manuscript and submitted a new manuscript.

Thanks again for the comments and valuable suggestions to improve our manuscript.

Kind regards,

Shusheng Yin.
