# Supplementary material for: Relationship between urban morphology and land surface temperature—A case study of Nanjing City
Source: PLoS One. 2022 Feb 9;17(2):e0260205. doi: 10.1371/journal.pone.0260205 (PMC8827472; doi:10.1371/journal.pone.0260205)
Supplement: Attachment — Submitted filename: Response to Reviewers(First).docx [file pone.0260205.s003.docx]

Dear Professor Yang,

Thank you very much for your letter, and the referees’ reports. Based on your comment and request, we have made extensive modification on the original manuscript. Here, we attached the revised manuscript in the formats of both manuscript and editable words for your approval. A document answering every question from the referees was also summarized and enclosed.

A revised manuscript with the correction sections blue marked was attached as the supplemental material and for easy check and editing purpose. If you have any questions, please contact us without hesitation.

**Reviewer 1:**

**Comment 1:** **This paper is overall interesting. However, the major revisions are required in terms of English, references, logic and structure.**

**Answers to comment 1:**

Thank you very much for referees’ reports. Based on your comments, I have made a lot of revisions to the article to ensure that the manuscript meets the publication requirements in terms of logic, structure, and references.

**Reviewer 2:**

**Comment 1:** **Since the purpose of this paper is to compare MGWR to GWR, it would be useful if the authors could expand this section to provide a bit more context. Has MGWR been applied to similar problems elsewhere? What is the theoretical motivation for applying MGWR to the challenge of LST prediction in an urban environment?**

**Answers to comment 1:**

Thank you very much for referees’ reports. First of all, the existing multi-scale geographic weighted regression models are mostly applied to urban rents, land prices, air pollution, etc., and urban thermal environment, as an important indicator of urban human settlements, has not been paid attention to in existing research. The manuscript applies MGWR to this research for the first time, with the expectation to analyze the urban thermal environmental impact mechanism from a more diverse perspective, and to provide a certain reference for mitigating the urban heat island effect.

Second, a large number of empirical studies have shown that there is significant spatial heterogeneity in the impact mechanism of urban thermal environment. Although the GWR model can solve the problem of spatial heterogeneity that traditional linear regression models cannot deal with to a certain extent, it ignores the difference. The difference in the spatial heterogeneity of the influencing factors leads to large estimation errors. MGWR improves the classical geographically weighted regression by allowing the bandwidth of each variable to be different, and then obtains a more credible estimation result, and gives different variables at the same time. The scale of influence.

**Comment 2:** **The first long paragraph is mostly unnecessary b/c the results are summarized in a table. I suggest that the authors remove this paragraph in favor of a short paragraph that highlights any particularly interesting features of these results. A similar comment applies to the Section 4.2 text that presents the numbers preported in Tables 4 and 5.**

**Answers to comment 2:**

Thank you very much for referees’ reports. This section mainly describes the spatial distribution and differences of LST in the study area, and its purpose is to lay the foundation for follow-up research. On the basis of your revised comments, we have reorganized the content of this section and rewritten this part.

**Amendment:**

### 4.1 Spatial pattern characteristics of LST

Using Landsat 8 images, the overall distribution of LST was inversed according to the single window algorithm (Fig. 2). The highest LST of Nanjing was 37.121 ℃, the lowest was 19.923 ℃, and the average was 28.525 ℃. According to an analysis of hot spots, excluding outliers and the spatial distribution of LST As shown in Figure 3.

Table 3 Statistics of LST in each district

| District | Minimum Temperature | Maximum Temperature | Cold spot | Proportion | Hot spot | Proportion |
| --- | --- | --- | --- | --- | --- | --- |
| Xuanwu | 22.978 | 31.570 | 413*** | 0.074 | 2172*** | 0.391 |
| Yuhuatai | 22.924 | 34.608 | 98*** | 0.030 | 331*** | 0.103 |
| Qinhuai | 24.506 | 31.744 | 24* | 0.006 | Not significant | Not significant |
| Gulou | 22.856 | 30.141 | 39*** | 0.008 | Not significant | Not significant |
| Jianye | 22.887 | 33.677 | 169*** | 0.047 | Not significant | Not significant |
| Qixia | 19.923 | 37.121 | 61*** | 0.030 | 838** | 0.410 |
| Pukou | 22.845 | 35.846 | 481*** | 0.106 | 2771** | 0.609 |
| Luhe | 20.666 | 33.437 | 927*** | 0.147 | 3452 | 0.550 |
| Lishui | 20.204 | 33.871 | 694*** | 0.131 | 3323** | 0.659 |
| Jiangning | 21.724 | 36.274 | 761*** | 0.163 | 2056*** | 0.441 |
| Gaochun | 21.104 | 35.072 | 417*** | 0.119 | 1681*** | 0.48 |

***indicates a confidence level of 0.99 , ** indicates a confidence level of 0.95, * indicates a confidence level of 0.90.

Overall, hotspots in Nanjing are concentrated mainly in Xuanwu District, Qixia District, Pukou District, Lishui District, Jiangning District, and Gaochun District. The highest value of LST was 37.12 ℃ in Qixia District, over an area located near the Lingang Industrial Concentration Zone in Qixia District and Nanjing Economic and Technological Development Zone. The area along the river between the two zones is largely covered by industrial land and residential land, with little vegetation. At the same time, the lowest value of LST in Nanjing was also observed in Qixia District, specifically in the Qixia Mountain. The area lies 268 m above sea level. The land use type is woodland, and the vegetation coverage is relatively high, with less interference from human activities. The distribution characteristics of LST hotspots in other areas are related to the cluster distribution of urban land, rural residential areas and other construction land, and the scattered distribution of waters and forests and grasslands, and are strongly affected by human activities.

Fig.2 Spatial distribution of LST in Nanjing

Fig.3 Hot spot analysis of LST

**Comment 3: Is MGWR better than GWR by a statistically significant amount? A test of statistical significance could be added to "Table 4" of this section (which I think is actually Table 7 in the manuscript).**

**Answers to comment 3:** Thank you for the mistakes you pointed out. The form has been checked.

I have made the explanation appropriate in the **4.3** section about verifying MGER and GWR.

**Amendment:**

Table 7 shows that the goodness of fit (R^2^) of MGWR is slightly higher than that of the precision GWR model, and the value of the corrected Akaike information criterion (AICc) is also lower than that of the classic GWR model. Therefore, MGWR can be assessed to have higher performance than the classic GWR. Comparing the residual sum of squares, the value of MGWR was also smaller than that of GWR. Moreover, MGWR requires fewer parameters to obtain a regression result closer to the true value.

Table 7 Model index of GWR and MGWR

| Model | MGWR | GWR |
| --- | --- | --- |
| R^2^ | 0.928 | 0.883 |
| AIC_C_ | 1303 | 1186 |
| Adj. R^2^ | 0.902 | 0.875 |
| Residual sum of squares | 1323 | 1977 |
| Sig. | 0.000* | 0.000* |

* Indicates that the test passed at the 5% significance level.

**Comment 4: Are the R2 statistics provided here for data that were used in fitting the MGWR and GWR, or for holdout data? While AIC is useful, it would be better to see how the models compare in out-of-sample prediction. Also, why is R2 and adjR2 the only metric used? What about MSE, or some other indicator of magnitude of error?**

**Answers to comment 4:**

Thank you very much for referees’ reports. It can be seen from Table 7 that the goodness of fit R2 of MGWR is higher than that of classic GWR, and the value of AICc is lower than that of classic GWR. From this, it can be determined that the result of MGWR is better than classic GWR. In terms of the number of effective parameters, the MGWR is smaller and the residual sum of squares is also smaller, indicating that it uses fewer parameters to obtain a regression result closer to the true value. Therefore, at least based on the analysis results of this case, it is found that the MGWR model is superior Based on the classic GWR model.

In addition, by reading a lot of existing literature, especially the literature on the MGWR model, the relevant literature is verified with parameters such as R2, AIC, Adj. R2, and the results of MSE are not used. Furthermore, this article is based on the existing research point of view, if the difference between the AICC values of the two models is greater than 3, the model with the lower AICC value will be regarded as the more model.

**Comment 5:** **It is unusual to have a "Conclusions" section between Results and Discussion. Perhaps this could be restructured to be a Section 4.4 "Summary" of results?**

**Answers to comment 5:**

Thank you very much for referees’ reports. This section has been modified. This chapter mainly intends to illustrate the limitations of the manuscript in research methods and research perspectives, with a view to solving them in future research.
